# Finite element analysis of maxillary orthodontic therapies with variable alveolar bone grafts under occlusal forces in patient with unilateral cleft lip and palate

**DOI:** 10.3389/fbioe.2024.1448286

**Published:** 2024-11-05

**Authors:** Zhi Zhang, Chenghao Li, Qian Zheng, Bing Shi, Renkai Liu

**Affiliations:** ^1^ State Key Laboratory of Oral Diseases, National Clinical Research Center for Oral Diseases, Department of Cleft Lip and Palate Surgery, West China Hospital of Stomatology, Sichuan University, Chengdu, China; ^2^ State Key Laboratory of Oral Diseases, National Clinical Research Center for Oral Diseases, Department of Pediatric dentistry, West China Hospital of Stomatology, Sichuan University, Chengdu, China

**Keywords:** unilateral cleft lip and palate, alveolar bone grafting, functional appliances, orthodontic treatment, occlusal forces, finite element analysis

## Abstract

**Objective:**

To investigate the biomechanical effects of maxillary orthodontic treatment on different alveolar bone grafting positions loaded with occlusal forces in an unilateral cleft lip and palate (UCLP) patient.

**Methods:**

Finite element analysis was employed to simulate clinical scenarios more accurately by loading with occlusal forces on 8 bone-grafted models during maxillary orthodontic treatment. Displacement and von Mises stress pattern during maxillary protraction, expansion, and combined protraction and expansion were analyzed.

**Results:**

The seven bone-grafted models exhibited significantly smaller horizontal displacements at the non-cleft side landmarks during maxillary protraction and expansion compared to non-bone grafted models. Additionally, alveolar cleft bone grafted in the upper 1/3 and middle 1/3 exhibited greater asymmetry displacement and stress under maxillary protraction and expansion.

**Conclusion:**

The study highlights the necessity of considering occlusal forces in finite element study on orthodontic therapies for UCLP patients. The upper 1/3 and middle 1/3 bone graft conditions may require secondary bone graft supplementation to ensure the effectiveness of maxillary orthodontic treatment.

## 1 Introduction

Alveolar clefts, as one of the most common congenital craniofacial defect, often lead to significant maxillary underdevelopment due to congenital jaw defects and scarring from facial and palatal surgery (Toro-Ibacache et al., 2014). This underdevelopment can result in a narrowed dental arch and a Class III skeletal profile, severely impacting the patient’s appearance and psychosocial wellbeing (Nucci et al., 2021; Mossey et al., 2009; Shi and Losee, 2015). Unilateral cleft lip and palate (UCLP) patients are particularly affected as a result of the asymmetry of the maxillary bones on both sides (Kochhar et al., 2021). Currently, the rectification of these maxillary deformities relies on a team approach, most crucially the surgical intervention of alveolar bone grafting (ABG) by surgeons and orthodontic interventions by orthodontists. ABG can provide continuity and stability to the maxilla, facilitating better orthodontic outcomes (Daw and Patel, 2004). However, bone resorption post-ABG remains a significant challenge, with reported resorption rates ranging from 10.4% to 100% (Jing et al., 2024; Tai et al., 2000; Zhang et al., 2012; Feichtinger et al., 2007). Therefore, understanding the biomechanical effects of functional orthodontics under varying bone graft volumes post-surgery is crucial for surgical planning, including potential secondary grafting, and offers predictive insights for orthodontic outcomes.

Finite element analysis remains the most dominating method to study biomechanical effect of maxillary functional orthodontic treatment, which mainly includes protraction and maxillary expansion, since it was first used in dental research in nearly 50 years ago (Thresher and Saito, 1973). However, with the increase of clinical demand, more simulated finite element models are needed to provide more accurate guidance for clinical practice (Grassia et al., 2024). Previous similar studies on maxillary therapies of UCLP have been primarily studied with an emphasis on maxillary protraction, while often neglecting the impact of occlusal forces, may result in potential shortcomings in the conclusions drawn (Chen et al., 2013; Chen et al., 2015; Parveen et al., 2020). Regarding maxillary expansion, existing research has predominantly focused on the timing of the procedure, whether pre-ABG or post-ABG (Velez-Muriel et al., 2021; Mathew et al., 2016). However, the specific effects of maxillary expansion on varying regional bone formation in UCLP have not been adequately investigated.

Recent studies have demonstrated that both occlusal and orthodontic forces significantly affect facial symmetry. Patients with alveolar clefts exhibit more asymmetric deformations under mastication (Luo et al., 2019). Meanwhile, magnitude of masticatory forces could affect the mechanical environment of sutures, with variations in strain magnitude, frequency, and the type of stress accelerating suture growth (Mao et al., 2003; Gautam et al., 2009; Mao, 2002). Our previous study also demonstrated that occlusal states were non-negligible for evaluating the stability of different bone graft types in UCLP (Tian et al., 2022). All of these illustrated that occlusal force should be included in the biomechanical study of functional orthodontics for alveolar cleft.

In this study, to more accurately simulate clinical conditions, we investigated biomechanical patterns in different graft types in an UCLP patient undergoing maxillary expansion, protraction, and combined protraction and expansion. This was done while applying occlusal forces and accurately constructing simulation models. This study was designed to elucidate the impact of bone formation at specific alveolar sites on the stability of various orthodontic treatments and to determine the conditions under which bone grafting may compromise the effectiveness of functional orthodontics, thereby necessitating supplementary grafting.

## 2 Materials and methods

### 2.1 Patient and equipment

An 11-year-old male patient, diagnosed with unilateral cleft lip and palate accompanied by maxillary hypoplasia was selected in this study, devoid of systemic bone diseases, periodontal disease or historical treatment of maxillary orthopedic and orthodontics. With the full informed consent of the patient and his parents, The patient’s head and neck region was scanned by Philips MX 16-slice X-ray computed tomography device (Philips Electronics, Netherlands) and CT data in DICOM (Digital Imaging and Communications in Medicine) format were collected for further analysis. The specific index was as follows: window width, 508*660 mm, bulb voltage, 120 kV, bulb current, 282 mA, obtained a total of 308 pieces with layer thickness of 1 mm, and layer spacing is 0.5 mm. All procedures were approved by the Medical Ethics Committee of West China Stomatology Hospital, Sichuan University (approval number: WCHSIRB-D-2022-001).

### 2.2 Establishment of UCLP maxillofacial 3D CAD models of non-bone graft

**FIGURE 1:**
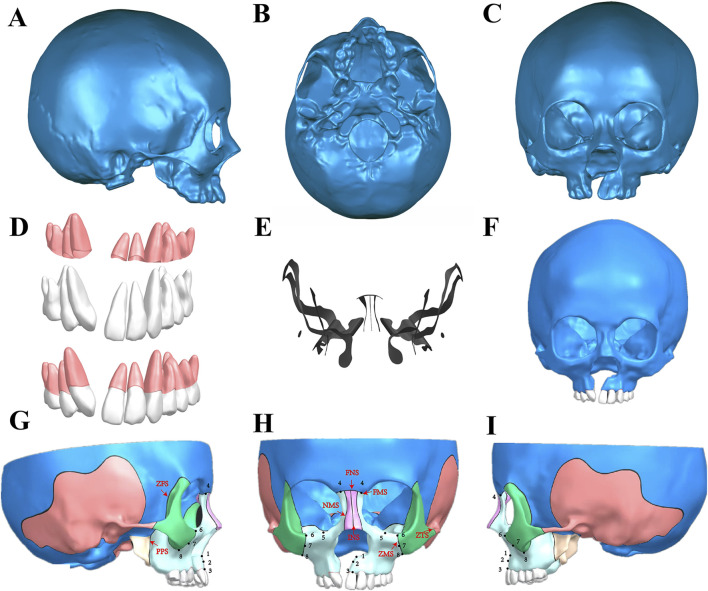
Establishment of UCLP craniomaxillofacial three-dimensional (3D) CAD model. **(A-C)** The preliminary model was obtained by repairing pores and trimming the surfaces of teeth and bones in Mimics; **(D)** Construction of maxillary teeth and periodontal ligaments; **(E)** Maxillary bone sutures; **(F)** A preliminary 3D model obtained by the further advanced refinements, including patching, smoothing, and detailed modeling of planes and surfaces were processed in Geomagic Studio; **(G-I)** Assembly of the maxillary teeth, the periodontal ligaments and the craniomaxillofacial bones in Siemens NX, showcasing the final UCLP maxillofacial 3D CAD model of non-bone graft and the selected landmarks of the maxilla and bone sutures: Frontal **(H)** and lateral view (Right: **(G)**, Left: **(I)**) of the model including selected landmarks of the maxilla and bone sutures. Landmarks: point 1, anterior nasal spine; point 2, subspinale; point 3, superior prosthion; point 4, frontal process; point 5, inferior orbital rim; point 6-8, superior, middle, and inferior zygomatic process. Bone sutures: the frontonasal (FNS), frontomaxillary (FMS), internasal (INS), frontozygomatic (ZFS), nasomaxillary (NMS), pterygopalatine (PPS), zygomaticomaxillary (ZMS), and zygomaticotemporal (ZTS) sutures.

CT data in DICOM format was imported into Mimics 20 software (Materialise, Belgium), employing Hounsfield Unit (HU) values pre-set to differentiate between bone and teeth (DenOtter and Schubert, 2021). Superfluous structures, including the mandibular cervical vertebra and the hyoid bone, were omitted to focus on the UCLP craniomaxillofacial complex. The resulting model underwent a series of refinements to repair pores and trim the surfaces of teeth and bones, and was saved (Figures 1A–C) in STL (StereoLithography) format. Further advanced refinements, including patching, smoothing, and detailed modeling of planes and surfaces were processed in Geomagic Studio 2014 (3D Systems, United States) to obtain a preliminary three-dimensional (3D) model in STEP (Standard for the Exchange of Product Model Data) format (Figure 1F). Concurrently, the teeth and periodontal membranes, with a thickness of 0.2 mm were reconstructed (Figure 1D) (Park et al., 2017; Tian et al., 2022). The UCLP 3D model, including the teeth and periodontal ligaments, was then imported into Siemens PLM NX 12.0.0 software (Siemens, Germany) for assembly, where further detailing was conducted. Bone sutures of 0.2 mm width, including the frontonasal (FNS), frontomaxillary (FMS), internasal (INS), frontozygomatic (ZFS), nasomaxillary (NMS), pterygopalatine (PPS), zygomaticomaxillary (ZMS), and zygomaticotemporal (ZTS) sutures, were accurately depicted (Figure 1E) (Knaup et al, 2004; Trojan et al., 2013; Fricke-Zech et al., 2012). The final UCLP craniomaxillofacial complex model was archived in PRT (Pro/ENGINEER) format, showcasing a detailed non-bone graft model (Figures 1G–I).

### 2.3 Establishment of 3D bone graft models in different sites of the alveolar cleft

**FIGURE 2:**
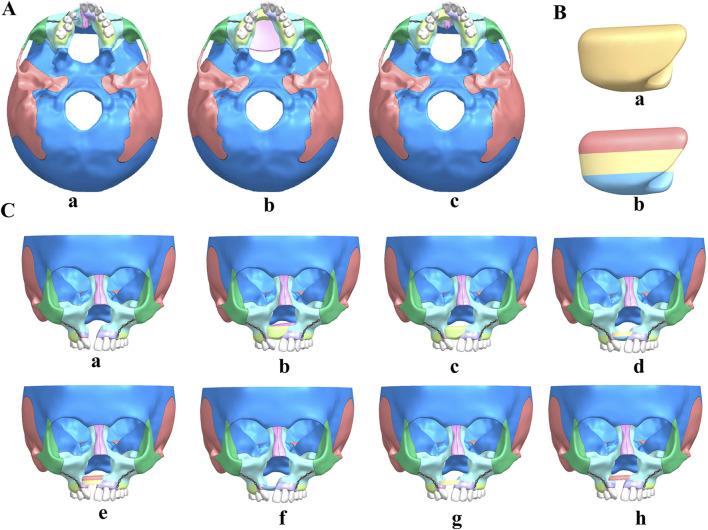
UCLP maxillofacial 3D models of non-bone graft and alveolar cleft bone graft. **(A)** non-bone graft **(a)**, full maxilla cleft **(b)** and full alveolar cleft **(c)** bone graft 3D model. **(B)** the bone module implanted in the alveolar cleft. **(C)** Maxillofacial 3D models of non-bone graft **(a)**, full maxilla cleft **(b)** and full alveolar cleft **(c)** bone graft, bone graft in lower 2/3 **(d)**, upper 2/3 **(e)**, lower 1/3 **(f)**, middle 1/3 **(g)** and upper 1/3 **(h)** of the alveolar cleft.

In this study, we employed Siemens NX software to create and analyze detailed 3D finite element models of cleft alveolar bone grafts, focusing on both total maxillary and alveolar clefts. The initial non-bone graft model was imported into the software, facilitating the generation of comprehensive models for full maxillary cleft bone grafts and full alveolar cleft bone grafts, as illustrated in Figure 2A.

Subsequently, the bone module implanted in the alveolar cleft, extracted from the 3D model of the full alveolar cleft bone graft (Figure 2Ba), was re-imported into Siemens NX. This module was divided into three equal sections based on height, with the alveolar crest designated as the lower section and the nasal base as the upper section, as depicted in Figure 2Bb. Considering various clinical scenarios of bone resorption, the bone fragments were segmented into five distinct grafting modes: upper 2/3, lower 2/3, upper 1/3, middle 1/3, and lower 1/3. These segments were then reassembled in Siemens NX software (Figure 2C) to construct corresponding bone grafting models. This methodological approach resulted in the formation of eight distinct UCLP 3D models for further analysis.

### 2.4 Boundary conditions and force loading

**FIGURE 3:**
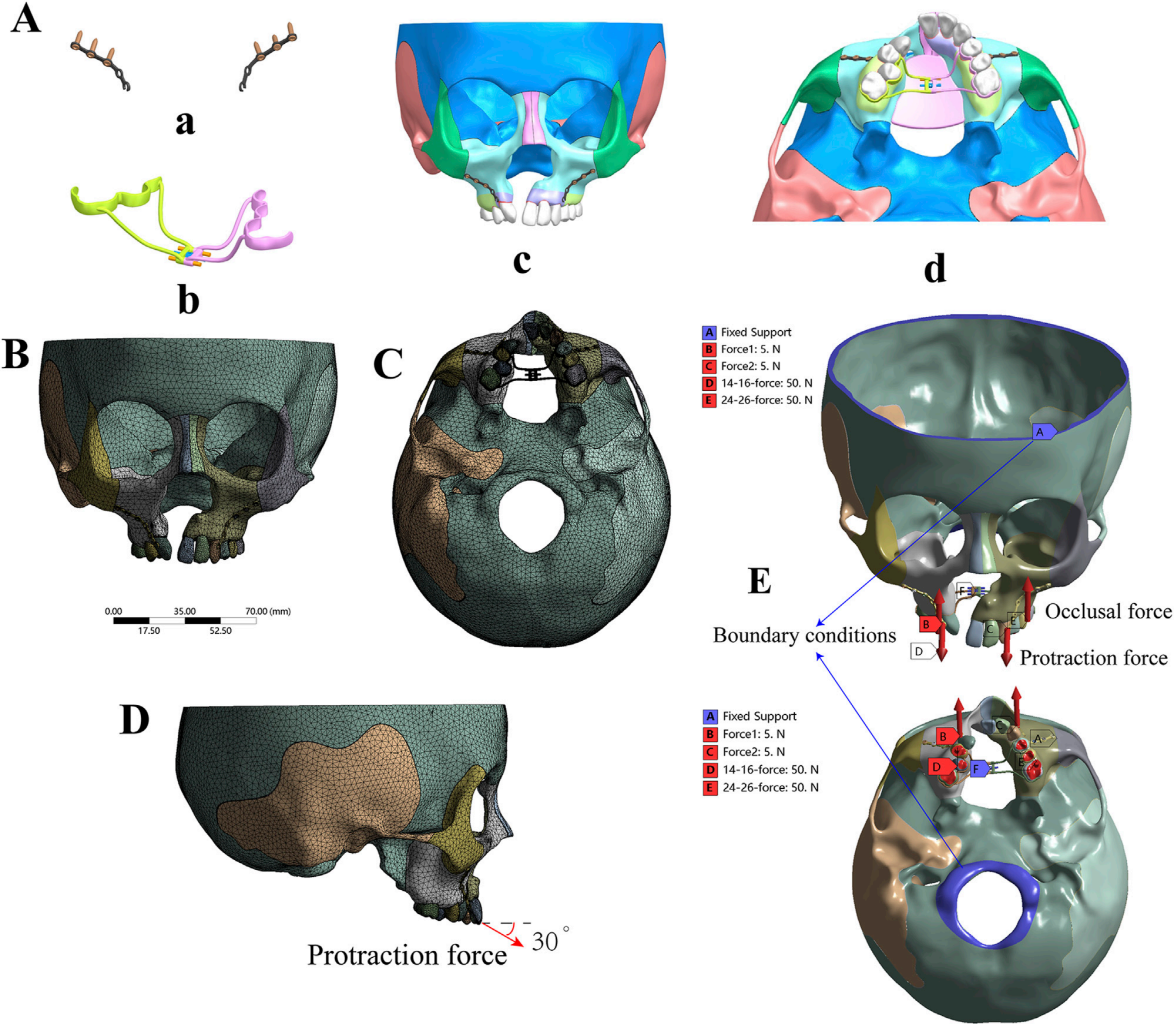
Boundary conditions and force loading. **(A)** Three-dimensional model of the miniplate **(a)**, hyrax spiral expander **(b)**, and the assembly of the UCLP 3D model **(c, d)**; **(B, C)** Tetrahedral meshing result of non-bone graft model with expander. **(D)** The direction of protraction force **(E)** Blue areas indicate fixed areas, which are the boundary areas (shown by blue arrows). Red arrows designate forces, including occlusal force [shown as red in **(B)** and **(C)**] and protraction force [shown as red in **(D)** and **(E)**].

In this study, ANSYS v19.2 Workbench software (ANSYS, United States) was utilized to create and analyze eight 3D finite element models of the UCLP cranio-maxillofacial complex under various mechanical loads to simulate clinical conditions. A conventional Hygienic Rapid Expander (hyrax) spiral rapid palatal expander was created via laser scanning and programmed as a finite element model for further study (Mathew et al., 2016), as illustrated in Figure 3Ab. Young’s modulus and Poisson’s ratios of structures were set according to Table 1 (Yu et al., 2007; Chen et al., 2015; Tanaka et al., 2016; Yoshida et al., 2001; Jones et al., 2001; Kim et al., 2015). The contact relationships of all adjacent structures were set to “bonded”. 10-node solid 187 elements (tetrahedron) were used for meshing. Mesh convergence was evaluated according to the results of the maximum displacement and the maximum principal stress (MPS) of the models under the simulated forces, following the general rules of FEM using (Schmidt et al., 2009). The most suitable convergence effect was obtained when the sizes of 0.5 mm, 1 mm, 0.3 mm, and 3 mm were used for tetrahedral meshes of periodontal ligaments and bone sutures, teeth and miniplates, expanders, and bones of the model, respectively. These models consisted of 405,515–497,447 elements and 795,106–948,728 nodes (Supplementary Table S3), and the tetrahedral meshing results of the models were shown in Supplementary Figure S1. One of the tetrahedral meshing results was shown in Figures 3B, C.

**TABLE 1:** Young’s modulus and Poisson’s ratio for the materials used in the models.

Material	Young’s modulus (MPa)	Poisson’s ratio
Cortical bone	13,700	0.3
Cancellous bone	7,900	0.3
Suture	0.68	0.47
Periodontal ligaments	0.69	0.49
Tooth	20,700	0.3
Miniplate and Miniscrew	103,000	0.33
Stainless steel	200,000	0.3

In addition, boundary conditions were applied to the nodes around the foramen magnum and frontal region of all models to mimic the stabilizing effect of a clinical mask structure, with zero displacement and rotation (Chen et al., 2013; Kumar et al., 2016; Velez-Muriel et al., 2021) as shown in blue in Figure 3E. All of the models were subjected to occlusal forces to replicate the state of maxillary dentition in centric relation occlusion, applying a force of 50N on the posterior teeth of both sides perpendicular to the occlusal plane (Velez-Muriel et al., 2021; Trojan et al., 2013; Widmark et al., 1995), as shown in Figure 3E. To simulate the protraction effect, a force of 5N was applied at the mesial ends of the bilateral titanium plates on the infrazygomatic crest, corresponding to a clinical protraction force of 500 g (Eom et al., 2018; Yang I. H. et al., 2012; Chen et al., 2013). This force was directed forward and downward at a 30° angle to the occlusal plane, as shown in Figure 3D. In terms of maxillary expansion, the hyrax expander, wildly employed to widen the dental arch in clinic practice (Park et al., 2017), exerts a distance of 0.25 mm per turn during expansion (Velez-Muriel et al., 2021), the expander force was applied at the lingual sides of the crowns of the first molar, first premolar, and second premolar (Parveen et al., 2020). The working conditions were classified into three groups, consisting of 24 models (Supplementary Figure S1), in order to simulate different clinical scenarios, protraction without expansion, expansion only and protraction with expansion of different bone graft types.

### 2.5 Analysis index

The analysis focused on two key indices: displacement and von Mises stress patterns. Particular attention was given to the bone sutures closely associated with the maxilla, as these play a critical role in facilitating three-dimensional maxillary expansion during functional orthodontic treatment. The von Mises stress in each suture was measured and compared across different groups using ANSYS Workbench software. This stress, also known as equivalent stress, represents the internal force that resists external pressures and returns the structure to its original shape after deformation (Lee and Baek, 2012; Yang I. H. et al., 2012; Chen et al., 2013). Simultaneously, we assessed the displacement of key maxillary landmarks across various models under conditions of protraction and expansion. The analysis primarily targeted the maxilla, which is significantly affected by functional orthodontics (Lee and Baek, 2012) (Figures 1G–I).

## 3 Results

### 3.1 Displacement and stress pattern on protraction without expansion

**FIGURE 4:**
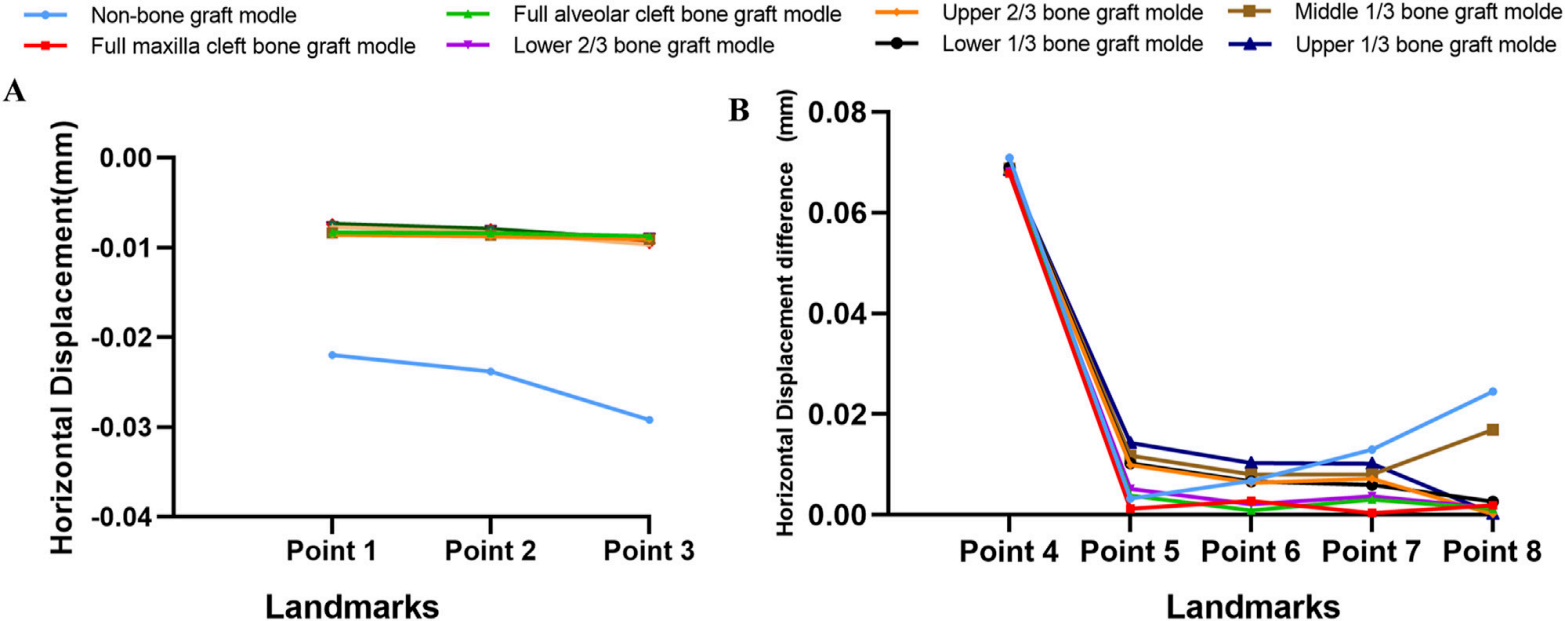
Displacement pattern on protraction without expansion. **(A)** Horizontal displacement of landmarks near the middle line in all models. **(B)** The difference of horizontal displacements of the landmarks between non-cleft sides and cleft sides.

Descriptive statistics of overall horizontal and sagittal displacement values of the selected landmarks after maxillary protraction without expansion were given in Supplementary Tables S1, S2. In terms of horizontal displacement, in the non-bone grafted model, horizontal displacement on the non-cleft side was greater than the cleft side. All seven bone-grafted models exhibited significantly reduced displacements on the non-cleft sides but notably increased displacements on the cleft sides of the maxilla compared to the non-bone graft model (Supplementary Table S1). Especially, the displacements of landmarks near the middle line were smaller in the seven bone-grafted model (Figure 4A). However, variations in sagittal displacement across different models were minimal (Supplementary Table S2). The asymmetry between the cleft and non-cleft sides under protraction is highlighted by the differences in horizontal displacements, with the non-bone graft model showing the largest disparity, followed by the upper 1/3 and middle 1/3 bone graft models. The lower 1/3 and upper 2/3 bone graft models showed intermediate differences, while the models with lower 2/3, full maxilla cleft, and full alveolar cleft bone grafts exhibited the smallest differences. Specifically, the displacement difference at the inferior point of the zygomatic process (point 8) was significantly greater in the non-bone graft and middle 1/3 bone graft models compared to the other models (Figure 4B).

**FIGURE 5:**
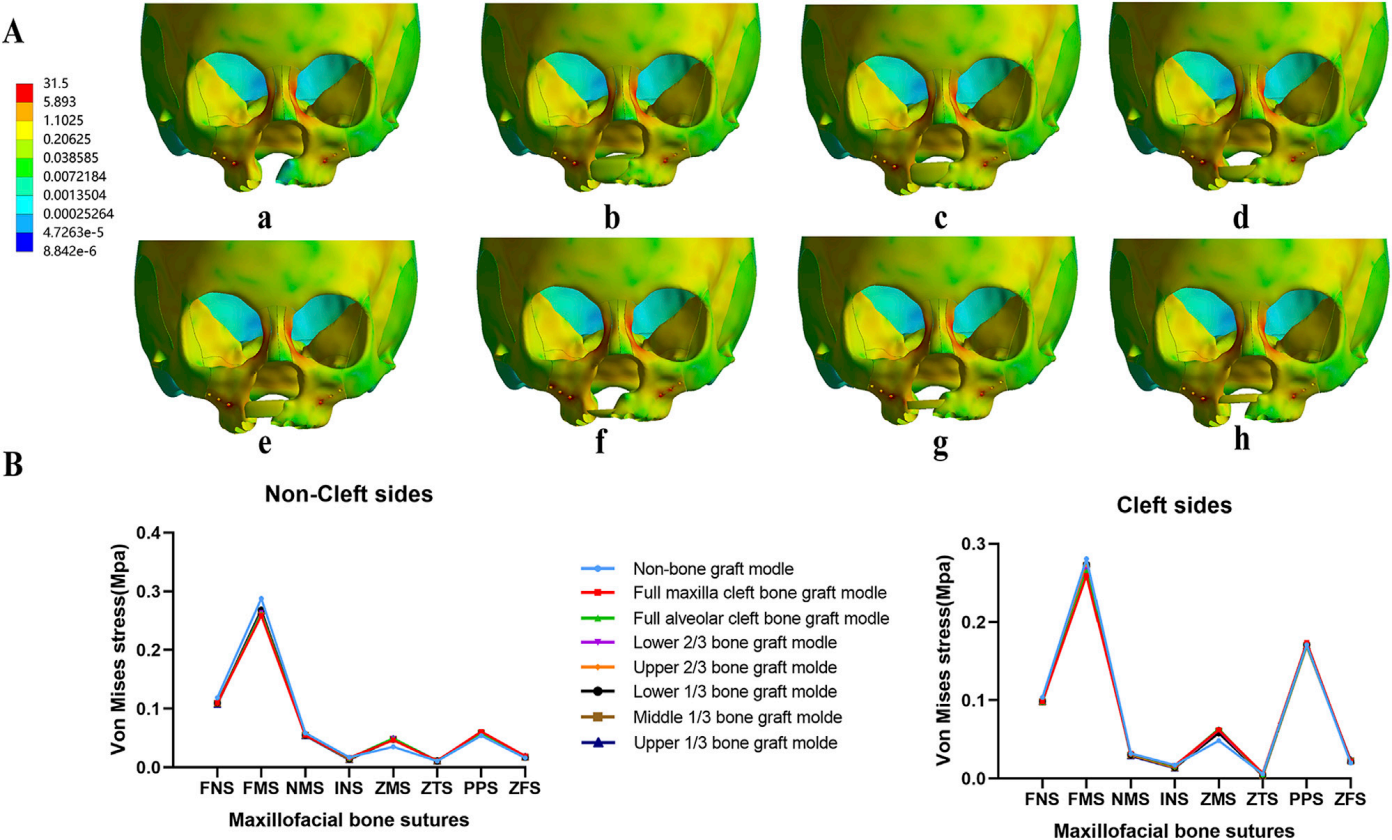
Von Mises stress distribution of maxillary complex and bone sutures under protraction. In all models, stress concentrated on frontal process of the maxilla **(A)**, and stress of all bone sutures show similar value in both non-cleft sides and cleft sides **(B)**.

Von Mises stress concentrated in the frontal process of the maxilla in all models with no notable differences among them (Figure 5A). The observed bone sutures showed no significant differences between the 8 models. However, von Mises stress at the pterygomaxillary suture (PPS) was significantly higher on the cleft side compared to the non-cleft side in all models (Figure 5B).

### 3.2 Displacement and stress pattern on expansion only

**FIGURE 6:**
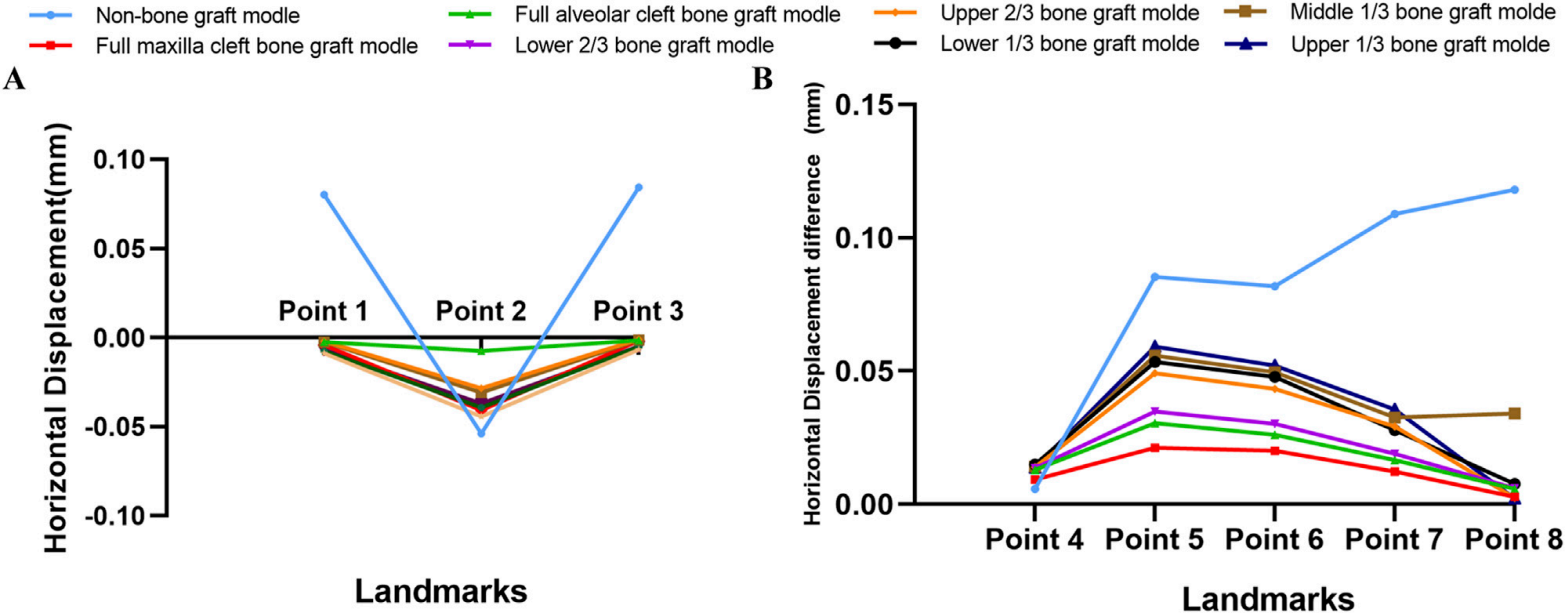
Displacement pattern on expansion. **(A)** Horizontal displacement of landmarks near the middle line in all models were obvious greater in non-bone graft model. **(B)** The difference of horizontal displacements of the landmarks between non-cleft sides and cleft sides.

Under maxillary expansion, horizontal displacements across different models were investigated. The seven bone-grafted models displayed significantly smaller horizontal displacements at the landmarks on both the cleft and non-cleft sides of the maxilla compared to the non-bone graft model. Moreover, the direction of displacement altered following bone grafting (Table 2). The displacements of landmarks near the middle line were small, and smaller in the seven bone graft model than non-bone graft model (Figure 6A). In comparison of symmetry on both sides, the non-bone graft model exhibited the largest disparity in horizontal displacement between the cleft and non-cleft sides, succeeded by the upper 1/3 and middle 1/3 bone graft models, then the lower 1/3 and upper 2/3 bone graft models. The models with lower 2/3 bone graft, full maxilla cleft bone graft, and full alveolar cleft bone graft demonstrated the smallest differences (Figure 6B).

**TABLE 2:** Horizontal(x) Displacement Value of the Landmarks in the 8 bone graft models under Maxillary Expansion (mm).

Selected landmarks		Non-bone graft	Full maxilla cleft	Full alveolar cleft	Lower 2/3	Upper 2/3	Lower 1/3	Middle 1/3	Upper 1/3
non-cleft side	**1**	8.02E-02	−2.35E-03	−2.06E-03	−2.50E-03	−6.75E-03	−4.18E-03	−7.50E-03	−8.86E-03
	**2**	−5.39E-02	−7.45E-03	−2.84E-02	−3.08E-02	−3.71E-02	−4.07E-02	−3.92E-02	−4.44E-02
	**3**	8.43E-02	−1.47E-03	−1.02E-03	−1.22E-03	−4.87E-03	−1.66E-03	−5.08E-03	−6.66E-03
	**4**	−2.61E-03	−4.09E-03	−3.98E-03	−4.22E-03	−4.01E-03	−5.32E-03	−4.68E-03	−3.48E-03
	**5**	4.01E-02	−1.08E-02	−8.65E-03	−1.08E-02	−1.67E-02	−2.12E-02	−2.10E-02	−2.00E-02
	**6**	4.02E-02	−9.80E-03	−6.65E-03	−8.55E-03	−1.39E-02	−1.80E-02	−1.78E-02	−1.67E-02
	**7**	5.40E-02	−5.95E-03	−2.19E-03	−3.24E-03	−7.19E-03	−8.46E-03	−9.39E-03	−8.81E-03
	**8**	5.89E-02	−1.55E-03	6.43E-03	6.53E-03	5.20E-03	6.37E-03	5.04E + 00	5.93E-03
cleft side	**4**	3.21E-03	5.21E-03	8.99E-03	9.32E-03	9.96E-03	9.66E-03	9.56E-03	1.03E-02
	**5**	−4.51E-02	1.05E-02	2.17E-02	2.40E-02	3.25E-02	3.22E-02	3.48E-02	3.92E-02
	**6**	−4.16E-02	1.03E-02	1.94E-02	2.16E-02	2.94E-02	2.98E-02	3.18E-02	3.54E-02
	**7**	−5.49E-02	6.35E-03	1.44E-02	1.57E-02	2.20E-02	1.93E-02	2.32E-02	2.68E-02
	**8**	−5.91E-02	1.28E-03	5.94E-04	4.93E-04	2.71E-03	−1.29E-03	2.56E-03	3.69E-03

Landmarks: point 1, anterior nasal spine; point 2, subspinale; point 3, superior prosthion; point 4, frontal process; point 5, inferior orbital rim; point 6–8, superior, middle, and inferior zygomatic process.

**FIGURE 7:**
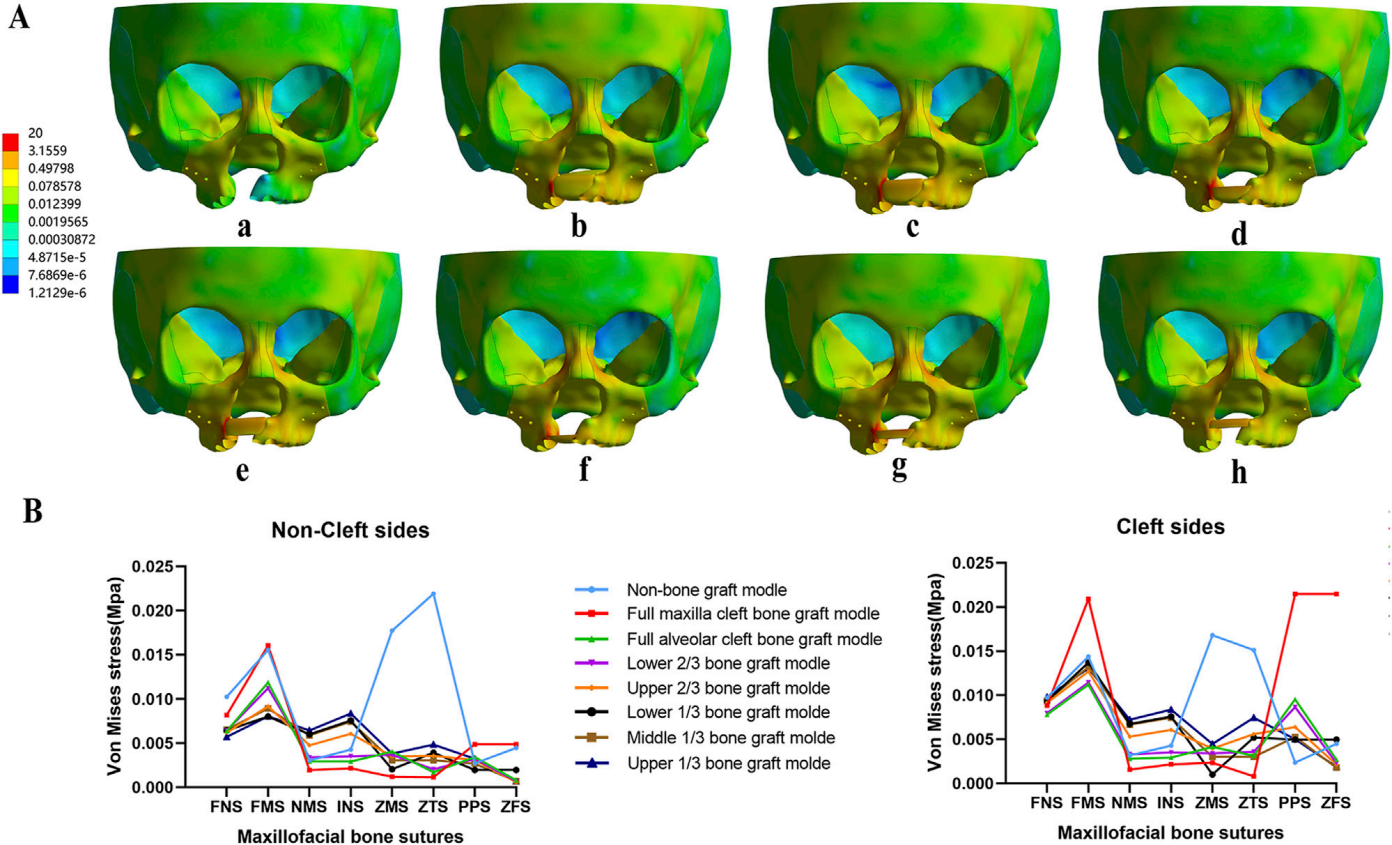
Von Mises stress distribution of maxillary complex and bone sutures under expansion. Stress mainly distributed on frontal process of the maxilla and implanted bone fragments in seven bone-grafted models **(A)**. Stress distribution of bone sutures in non-cleft sides and cleft sides **(B)**.

Regarding the distribution of von Mises stress during maxillary expansion, it was primarily located in the frontal process, alveolar process, and implanted bone mass of the maxilla. Among the implanted bone fragments, the von Mises stress was highest in the upper 1/3 and middle 1/3 bone graft models, with the upper 1/3 model experiencing the greatest stress (Figure 7A). Additionally, the von Mises stress in the zygomaticomaxillary suture (ZMS) was notably higher in these models than in other grafted models on both the cleft and non-cleft sides (Figure 7B).

### 3.3 Displacement and stress pattern on protraction with expansion

**FIGURE 8:**
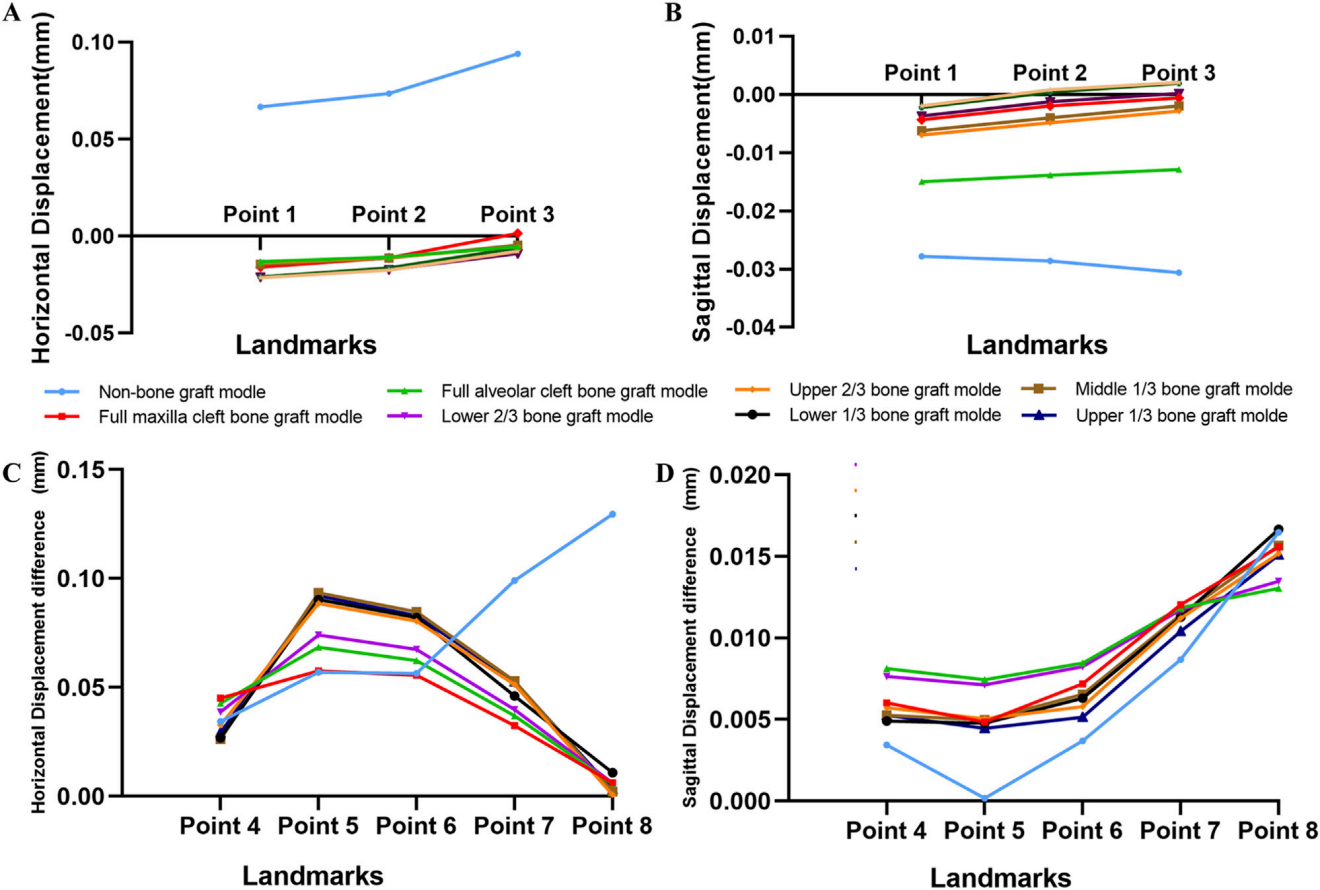
Displacement pattern on protraction and expansion. **(A)** Horizontal displacement of landmarks near the middle line in all models were obvious greater in non-bone graft model. **(B)** The difference of horizontal displacements of the landmarks between non-cleft sides and cleft sides. **(C)** Sagittal displacement of landmarks in all models. **(D)** The difference of sagittal displacements of the landmarks between non-cleft sides and cleft sides.

Under the combined forces of protraction and maxillary expansion, the seven bone graft models exhibited significantly smaller horizontal and sagittal displacements at the maxillary landmarks on both the non-cleft and cleft sides compared to the non-bone graft model (Table 3, 4). Both the horizontal and sagittal displacements of landmarks near the middle line were smaller in the seven bone graft model than non-bone graft model (Figures 8A,C). Among the bone graft models, the horizontal displacement differences were most pronounced in the upper 1/3, middle 1/3, lower 1/3, and upper 2/3 bone graft models, followed in magnitude by the lower 2/3, full alveolar cleft, and full maxilla cleft bone graft models (Figures 8B,D).

**TABLE 3:** Horizontal and Sagittal Displacement Value of the Landmarks in Non-cleft side of the 8 models under Maxillary Protraction and Expansion (mm).

Selected landmarks		Non-bone graft	Full maxilla cleft	Full alveolar cleft	Lower 2/3	Upper 2/3	Lower 1/3	Middle 1/3	Upper 1/3
horizontal(x)	**1**	6.67E-02	−1.31E-02	−1.37E-02	−1.45E-02	−2.10E-02	−1.60E-02	−2.10E-02	−2.15E-02
	**2**	7.35E-02	−1.08E-02	−1.10E-02	−1.14E-02	−1.72E-02	−1.12E-02	−1.64E-02	−1.75E-02
	**3**	9.40E-02	−5.43E-03	−5.45E-03	−4.56E-03	−8.92E-03	1.39E-03	−5.97E-03	−7.85E-03
	**4**	−2.54E-02	−3.33E-02	−3.16E-02	−2.99E-02	−2.93E-02	−2.59E-02	−2.53E-02	−2.76E-02
	**5**	1.00E-02	−3.53E-02	−3.46E-02	−3.81E-02	−4.45E-02	−4.99E-02	−4.92E-02	−4.60E-02
	**6**	1.30E-02	−3.28E-02	−3.05E-02	−3.37E-02	−3.95E-02	−4.42E-02	−4.36E-02	−4.05E-02
	**7**	3.59E-02	−2.17E-02	−1.83E-02	−2.02E-02	−2.50E-02	−2.60E-02	−2.72E-02	−2.51E-02
	**8**	5.46E-02	−8.86E-03	7.42E-04	4.87E-04	−1.80E-03	7.60E-04	−1.37E-03	2.25E-05
Sagittal(y)	**1**	−2.78E-02	−1.50E-02	−6.96E-03	−6.22E-03	−3.70E-03	−4.33E-03	−2.24E-03	−1.93E-03
	**2**	−2.86E-02	−1.39E-02	−4.84E-03	−4.00E-03	−1.24E-03	−1.97E-03	3.78E-04	8.15E-04
	**3**	−3.06E-02	−1.29E-02	−2.84E-03	−1.96E-03	1.91E-04	−5.80E-04	1.95E-03	2.05E-03
	**4**	−9.40E-03	−1.11E-02	−9.62E-03	−9.65E-03	−8.31E-03	−9.06E-03	−8.07E-03	−7.60E-03
	**5**	−2.22E-02	−2.48E-02	−3.11E-02	−3.18E-02	−3.24E-02	−3.37E-02	−3.32E-02	−3.34E-02
	**6**	−2.27E-02	−2.92E-02	−4.06E-02	−4.21E-02	−4.46E-02	−4.64E-02	−4.65E-02	−4.70E-02
	**7**	−2.23E-02	−2.93E-02	−4.10E-02	−4.22E-02	−4.57E-02	−4.64E-02	−4.70E-02	−4.80E-02
	**8**	−2.37E-02	−3.00E-02	−4.28E-02	−4.40E-02	−4.83E-02	−4.86E-02	−4.94E-02	−5.08E-02

Landmarks: point 1, anterior nasal spine; point 2, subspinale; point 3, superior prosthion; point 4, frontal process; point 5, inferior orbital rim; point 6–8, superior, middle, and inferior zygomatic process.

**TABLE 4:** Horizontal and Sagittal Displacement Value of the Landmarks in cleft side of the 8 models under Maxillary Protraction and Expansion (mm).

Selected landmarks		Non-bone graft	Full maxilla cleft	Full alveolar cleft	Lower 2/3	Upper 2/3	Lower 1/3	Middle 1/3	Upper 1/3
horizontal(x)	**4**	8.92E-03	1.18E-02	1.11E-02	8.84E-03	3.43E-03	8.93E-04	8.02E-04	1.53E-03
	**5**	−4.69E-02	2.22E-02	3.39E-02	3.60E-02	4.40E-02	4.05E-02	4.42E-02	4.61E-02
	**6**	−4.34E-02	2.28E-02	3.18E-02	3.38E-02	4.09E-02	3.79E-02	4.11E-02	4.25E-02
	**7**	−6.31E-02	1.07E-02	1.87E-02	1.97E-02	2.62E-02	2.01E-02	2.57E-02	2.75E-02
	**8**	−7.49E-02	−2.63E-03	−4.96E-03	−5.53E-03	−2.48E-03	−1.01E-02	−3.60E-03	−3.11E-03
Sagittal(y)	**4**	−5.95E-03	−5.03E-03	−1.49E-03	−2.00E-03	−2.58E-03	−4.16E-03	−2.80E-03	−2.34E-03
	**5**	−2.20E-02	−2.00E-02	−2.36E-02	−2.47E-02	−2.73E-02	−2.89E-02	−2.82E-02	−2.90E-02
	**6**	−1.90E-02	−2.20E-02	−3.21E-02	−3.38E-02	−3.88E-02	−4.00E-02	−3.99E-02	−4.18E-02
	**7**	−1.37E-02	−1.72E-02	−2.92E-02	−3.05E-02	−3.44E-02	−3.51E-02	−3.56E-02	−3.76E-02
	**8**	−7.18E-03	−1.44E-02	−2.98E-02	−3.05E-02	−3.31E-02	−3.19E-02	−3.37E-02	−3.57E-02

Landmarks: point 4, frontal process; point 5, inferior orbital rim; point 6–8, superior, middle, and inferior zygomatic process.

**FIGURE 9:**
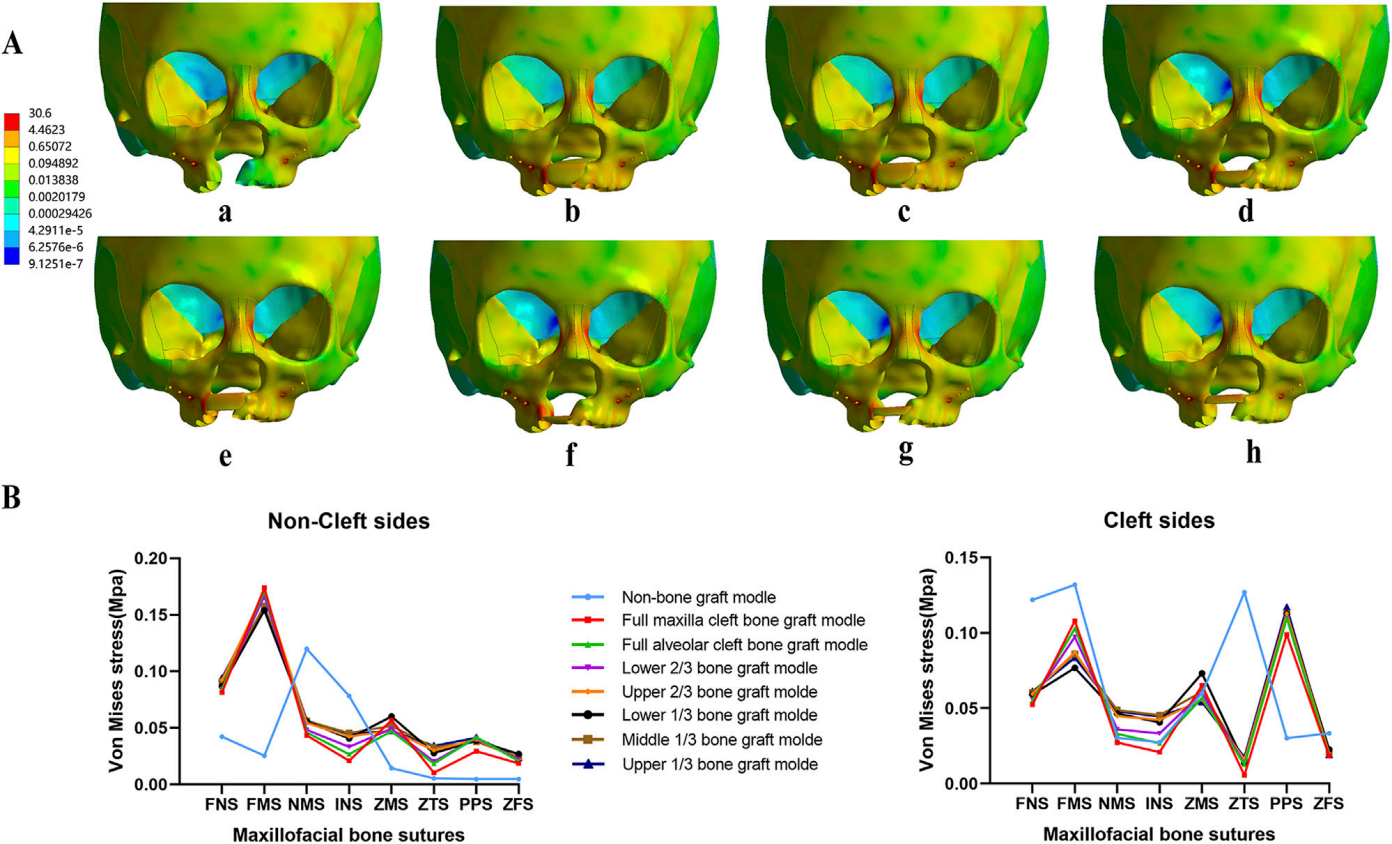
Von Mises stress distribution of maxillary complex and bone sutures under protraction and expansion. Stress on frontal process of the maxilla and implanted bone fragments were obvious greater in upper 1/3 bone graft models **(A)**. Stress distribution of bone sutures in non-cleft sides and cleft sides **(B)**.

Concurrently, the distribution of von Mises stress was primarily observed in the frontal process, alveolar process, and implanted bone mass of the maxillas. The von Mises stress in the frontal process of the maxilla was lower in the non-bone graft model compared to the bone graft models. Notably, the stress distribution in the non-bone graft model differed significantly from that in the bone graft models (Figure 9A). On the non-cleft side, the nasomaxillary suture (NMS) and infraorbital suture (INS) exhibited significantly higher stress levels compared to other bone graft models, whereas the frontomaxillary suture (FMS) showed significantly lower stress. On the cleft side, the FMS and zygomaticotemporal suture (ZTS) displayed significantly higher von Mises stress, while the pterygopalatine suture (PPS) showed lower stress levels. In the bone graft models, the upper 1/3, middle 1/3, lower 1/3, and upper 2/3 bone graft models experienced higher stress across each bone suture (Figure 9B).

## 4 Discussion

Distinguishing it from previous studies, this finite element analysis study incorporated occlusal force factors into the analysis of alveolar cleft bone grafting effects under maxillary orthodontic conditions. It was well-established that occlusal forces are essential for exploring orthodontic treatments of the maxilla. Chen et al. developed six finite element ABG models without loaded occlusal forces to simulate various types of bone resorption following alveolar bone grafting and subsequent protraction orthodontic treatment (Chen et al., 2013). Their findings indicate that non-resorptive models demonstrate optimal outcomes under maxillary protraction forces, with lower bone graft resorption showing superior protraction effects compared to upper bone graft resorption. However, the results of this study show that alveolar clefts grafted in the upper 1/3 and middle 1/3 are more unstable under protraction forces, differing from their results. This discrepancy may arise from the significant alterations in the magnitude and direction of protraction forces due to occlusal forces. Research shows that occlusal forces are distributed across five vertical and two horizontal supports in the midfacial skeleton. These forces, with potential changes in magnitude and/or direction, can lead to significant changes in cortical and trabecular bone structures (Janovic et al., 2015). It was noted that mechanical stress from natural activities (such as chewing) and external forces (such as orthodontic therapy) collectively influence mechanical transmission at sutures (Mao et al., 2003). Therefore, these results demonstrate again that the effect of occlusal force loading on maxillary orthodontic treatments is significant. Therefore, to accurately simulate clinical scenarios, more finite element analysis studies involved occlusal force are needed to illustrate the biomechanical effects of maxillary orthodontic post alveolar cleft bone graft. However, this study solely considered the occlusal forces of the centric occlusion. In further studies, we will explore the effects of occlusal forces in various occlusion scenarios to offer more precise guidance for clinical practice.

The accuracy of finite element analysis results largely depends on the accuracy of the modeling (Gautam et al., 2007). This research ensures precise data by using low-radiation CT scans, obtaining 1 mm slice thickness and 0.5 mm inter-slice spacing DICOM data, and utilizing various software to accurately reconstruct 3D models. Herring et al. emphasized studying craniofacial bones as composites of independent bone segments with sutures, rather than as a single structural entity, due to the unique structure of unilateral complete cleft lip and palate patients (Herring and Teng, 2000). This approach involves modeling eight key suture systems closely related to maxillary growth and orthodontic treatment (Lee and Baek, 2012; Yang I. H. et al., 2012) with suture widths set at 0.2 mm (Knaup et al., 2004; Fricke-Zech et al., 2012; Wang et al., 2009). The significant role of the periodontal ligament in stress distribution is well recognized (Tanne et al., 1987; Middleton et al., 1996), necessitating its reconstruction for accurate biomechanical behavior simulation during chewing, orthodontic treatment, and maxillary expansion. Thus, the study involves separating the dental arch, expanding it by 0.2 mm on its surface (Yoshida et al., 2001; Wang et al., 2009) to simulate the periodontal ligament and reassembling it into the craniofacial complex. Moreover, to closely mimic clinical maxillary expansion situations, differing from previous studies that applied direct force or displacement on either side of the dental arch, this study employed hyrax spiral expanders (Carvalho Trojan et al., 2017; Ngan et al., 2015; Velez-Muriel et al., 2021), where one complete turn of the expander equates to 0.25 mm expansion, effectively averaging 0.125 mm per side. These measures ensured the precision of the reconstruction of the alveolar bone graft models, providing a solid foundation for subsequent biomechanical analysis.

One main controversy in arch constriction for patients with alveolar clefts is whether to expand the dental arch before or after bone grafting. The prevailing view supports post-grafting maxillary expansion, as it is beneficial for reducing defect size, facilitating tension-free gingival closure, and requiring less bone graft material, thus promoting more unified maxillary bone and enabling palatal suture expansion (Santiago et al., 2014). Further clinical studies demonstrate that rapid maxillary expansion (RME) post alveolar grafting for UCLP does not compromise the graft’s effectiveness, regardless of RME success rates (da Silva Filho et al., 2009). Similarly, Ade et al. found that RME had no adverse effects on bilateral graft regions in BCLP patients, and confirmed the orthodontic effectiveness of suture expansion (Cavassan Ade et al., 2004). Yang et al. also conducted maxillary expansion in post-graft UCLP patients, with cephalometric analysis showing significant increases in maxillary and arch widths without notable radiographic changes in the grafted areas (Yang C. J. et al., 2012). These findings emphasize the significance of maxillary expansion in correcting arch constriction post-alveolar cleft bone grafting. However, bone resorption frequently occurs subsequent to alveolar bone grafting. It is clinically significant to investigate the biomechanical effects of maxillary orthodontic forces under varying osteogenic conditions.

The development of maxillary protraction techniques increasingly incorporates temporary anchorage devices, gaining attention for anterior maxillary protraction in patients with midfacial deficiencies. Miniplates placed below the zygoma as skeletal anchorage systems have proven effective in correcting Class III malocclusion. In contrast, palatal plates can be placed at a single site without flaps or incisions, minimizing risks to critical anatomical structures. Although studies found that greater anterior movement with palatal plates compared to zygomatic miniplates and traditional dental adjusters, the application of palatal plates in alveolar cleft is limited due to absence of palatal plate. Therefore, in this study, miniplates were placed on the zygomatic alveolar ridge. Regarding the magnitude of protraction forces, optimal force values should be minimal to achieve maximal skeletal effects and minimal dental effects. Yepes et al. recommend using forces of 300–400 g, as they produce similar effects without causing greater biological wear on the body (Yepes et al., 2014). Parveen et al. suggest that compared to facemask protraction forces (600 g), using protraction forces of 1200 g maintains initial displacement and stress distribution within a higher range (Parveen et al., 2020). For younger patients with ample treatment time and relatively fragile sutures, smaller forces of less than 5N per side are chosen to avoid compressing the upper dental arch and causing potential damage to the cranio-maxillary complex. For older patients with limited treatment time and gradually closing sutures, larger forces of more than 5N per side are selected to achieve treatment goals quickly. Therefore, 5N was set as the magnitude of protraction forces in this study.

Under maxillary protraction or expansion, the results presented highlight notable distinctions in displacement and stress patterns when comparing bone-grafted models to non-bone grafted models. The results demonstrate that bone-grafted models undergo significantly less horizontal displacement at both cleft and non-cleft side landmarks when subjected to maxillary protraction forces, compared to non-bone grafted counterparts. This suggests that bone grafting might stabilize the skeletal structure, thereby mitigating asymmetry between the cleft and non-cleft sides. The von Mises stress of implanted bone fragments was highly distributed under maxillary expansion forces, while it was small under the action of protraction or combined protraction and expansion forces. This suggests that protraction may be more conducive to the stability of the implanted bone, and combined maxillary protraction and expansion may be better than expansion alone in UCLP patients.

Notably, the study highlights significant variance in the displacement across different grafting models between non-cleft and cleft sides, with the upper 1/3 and middle 1/3 bone graft models showing the greatest difference. Although the observed bone sutures showed no significant differences between the 8 models under maxillary protraction. Under maxillary expansion, the von Mises stress in the zygomaticomaxillary suture (ZMS) was notably higher in the upper 1/3 and middle 1/3 bone graft models than in other grafted models on both the cleft and non-cleft sides. However, under maxillary protraction and expansion, the bone graft models, including the upper 1/3, middle 1/3, lower 1/3, and upper 2/3 bone graft models, experienced higher stress across each bone suture. All these results indicate that grafting in the upper 1/3 and middle 1/3 conditions may require secondary bone graft supplementation to ensure the effectiveness of maxillary orthodontic treatments.

## 5 Conclusion

This study underscored the critical importance of incorporating occlusal forces in finite element study on orthodontic therapies for UCLP patients. Under occlusal forces, the upper 1/3 and middle 1/3 bone graft conditions exhibited greater asymmetry between cleft and non-cleft sides during maxillary orthodontic treatment. This suggests that additional bone graft supplementation may be required to optimize the effectiveness of maxillary orthodontic treatments in these areas. Further research is expected to explore the effects of occlusal forces in various occlusion scenarios on the stability of the UCLP craniomaxillofacial complex, which could provide valuable clinical insights and refine treatment strategies for improved patient care.

## Data Availability

The original contributions presented in the study are included in the article/Supplementary Material, further inquiries can be directed to the corresponding authors.

## References

[B1] Carvalho Trojan L. Andres Gonzalez-Torres L. Claudia Moreira Melo A. Barbosa de Las Casas E. (2017). Stresses and strains analysis using different palatal expander appliances in upper jaw and midpalatal suture. Artif. Organs 41 (6), E41–E51. 10.1111/aor.12817 27925236

[B2] Cavassan Ade O. de Albuquerque M. D. Filho L. C. (2004). Rapid maxillary expansion after secondary alveolar bone graft in a patient with bilateral cleft lip and palate. Cleft Palate Craniofac J. 41 (3), 332–339. 10.1597/02-099.1 15151452

[B3] Chen Z. Pan X. Shao Q. Chen Z. (2013). Biomechanical effects on maxillary protraction of the craniofacial skeleton with cleft lip and palate after alveolar bone graft. J. Craniofac Surg. 24 (2), 446–453. 10.1097/SCS.0b013e31826cfe27 23524712

[B4] Chen Z. Pan X. Zhao N. Chen Z. Shen G. (2015). Asymmetric maxillary protraction for unilateral cleft lip and palate patients using finite element analysis. J. Craniofac Surg. 26 (2), 388–392. 10.1097/SCS.0000000000001337 25759916

[B5] da Silva Filho O. G. Boiani E. de Oliveira Cavassan A. Santamaria M. Jr. (2009). Rapid maxillary expansion after secondary alveolar bone grafting in patients with alveolar cleft. Cleft Palate Craniofac J. 46 (3), 331–338. 10.1597/07-205.1 19642749

[B6] Daw J. L. Jr. Patel P. K. (2004). Management of alveolar clefts. Clin. Plast. Surg. 31 (2), 303–313. 10.1016/S0094-1298(03)00129-9 15145671

[B7] DenOtter T. D. Schubert J. (2021). “Hounsfield unit,” in StatPearls (Treasure Island (FL): StatPearls Publishing). 31613501

[B8] Eom J. Bayome M. Park J. H. Lim H. J. Kook Y. A. Han S. H. (2018). Displacement and stress distribution of the maxillofacial complex during maxillary protraction using palatal plates: a three-dimensional finite element analysis. Korean J. Orthod. 48 (5), 304–315. 10.4041/kjod.2018.48.5.304 30206529 PMC6123076

[B9] Feichtinger M. Mossbock R. Karcher H. (2007). Assessment of bone resorption after secondary alveolar bone grafting using three-dimensional computed tomography: a three-year study. Cleft Palate Craniofac J. 44 (2), 142–148. 10.1597/06-047.1 17328652

[B10] Fricke-Zech S. Gruber R. M. Dullin C. Zapf A. Kramer F. J. Kubein-Meesenburg D. (2012). Measurement of the midpalatal suture width. Angle Orthod. 82 (1), 145–150. 10.2319/040311-238.1 21812573 PMC8881041

[B11] Gautam P. Valiathan A. Adhikari R. (2007). Stress and displacement patterns in the craniofacial skeleton with rapid maxillary expansion: a finite element method study. Am. J. Orthod. Dentofac. Orthop. 132 (1), 5 e1–e11. 10.1016/j.ajodo.2006.09.044 17628242

[B12] Gautam P. Valiathan A. Adhikari R. (2009). Skeletal response to maxillary protraction with and without maxillary expansion: a finite element study. Am. J. Orthod. Dentofac. Orthop. 135 (6), 723–728. 10.1016/j.ajodo.2007.06.016 19524831

[B13] Grassia V. d'Apuzzo F. Alansari R. A. Jamilian A. Sayahpour B. Adel S. M. (2024). Instagram and clear aligner therapy: a content analysis of patient perspectives. Seminars Orthod. 10.1053/j.sodo.2024.05.009

[B14] Herring S. W. Teng S. (2000). Strain in the braincase and its sutures during function. Am. J. Phys. Anthropol. 112 (4), 575–593. 10.1002/1096-8644(200008)112:4<575::AID-AJPA10>3.0.CO;2-0 10918130 PMC2813197

[B15] Janovic A. Saveljic I. Vukicevic A. Nikolic D. Rakocevic Z. Jovicic G. (2015). Occlusal load distribution through the cortical and trabecular bone of the human mid-facial skeleton in natural dentition: a three-dimensional finite element study. Ann. Anat. 197, 16–23. 10.1016/j.aanat.2014.09.002 25458179

[B16] Jing B. Yang C. Tsauo C. Low D. W. Tao H. Shi B. (2024). Evaluation of secondary alveolar bone grafting for unilateral complete cleft alveolus: a retrospective cone beam computed tomography-based study. Facial Plast. Surg. Aesthet. Med. 26, 564–570. 10.1089/fpsam.2023.0257 38621184

[B17] Jones M. L. Hickman J. Middleton J. Knox J. Volp C. (2001). A validated finite element method study of orthodontic tooth movement in the human subject. J. Orthod. 28 (1), 29–38. 10.1093/ortho/28.1.29 11254801

[B18] Kim K. Y. Bayome M. Park J. H. Kim K. B. Mo S. S. Kook Y. A. (2015). Displacement and stress distribution of the maxillofacial complex during maxillary protraction with buccal versus palatal plates: finite element analysis. Eur. J. Orthod. 37 (3), 275–283. 10.1093/ejo/cju039 25090997

[B19] Knaup B. Yildizhan F. Wehrbein H. (2004). Age-related changes in the midpalatal suture. A histomorphometric study. J. Orofac. Orthop. 65 (6), 467–474. 10.1007/s00056-004-0415-y 15570405

[B20] Kochhar A. S. Nucci L. Sidhu M. S. Prabhakar M. Grassia V. Perillo L. (2021). Reliability and reproducibility of landmark identification in unilateral cleft lip and palate patients: digital lateral vis-A-vis CBCT-derived 3D cephalograms. J. Clin. Med. 10 (3), 535. 10.3390/jcm10030535 33540549 PMC7867146

[B21] Kumar A. Ghafoor H. Khanam A. (2016). A comparison of three-dimensional stress distribution and displacement of naso-maxillary complex on application of forces using quad-helix and nickel titanium palatal expander 2 (NPE2): a FEM study. Prog. Orthod. 17 (1), 17. 10.1186/s40510-016-0131-3 27245236 PMC4896894

[B22] Lee N. K. Baek S. H. (2012). Stress and displacement between maxillary protraction with miniplates placed at the infrazygomatic crest and the lateral nasal wall: a 3-dimensional finite element analysis. Am. J. Orthod. Dentofac. Orthop. 141 (3), 345–351. 10.1016/j.ajodo.2011.07.021 22381495

[B23] Luo X. Huang H. Yin X. Shi B. Li J. (2019). Functional stability analyses of maxillofacial skeleton bearing cleft deformities. Sci. Rep. 9 (1), 4261. 10.1038/s41598-019-40478-w 30862870 PMC6414651

[B24] Mao J. J. (2002). Mechanobiology of craniofacial sutures. J. Dent. Res. 81 (12), 810–816. 10.1177/154405910208101203 12454093

[B25] Mao J. J. Wang X. Kopher R. A. (2003). Biomechanics of craniofacial sutures: orthopedic implications. Angle Orthod. 73 (2), 128–135. 10.1043/0003-3219(2003)73<128:BOCSOI>2.0.CO;2 12725368

[B26] Mathew A. Nagachandran K. S. Vijayalakshmi D. (2016). Stress and displacement pattern evaluation using two different palatal expanders in unilateral cleft lip and palate: a three-dimensional finite element analysis. Prog. Orthod. 17 (1), 38. 10.1186/s40510-016-0150-0 27800592 PMC5116441

[B27] Middleton J. Jones M. Wilson A. (1996). The role of the periodontal ligament in bone modeling: the initial development of a time-dependent finite element model. Am. J. Orthod. Dentofac. Orthop. 109 (2), 155–162. 10.1016/s0889-5406(96)70176-2 8638561

[B28] Mossey P. A. Little J. Munger R. G. Dixon M. J. Shaw W. C. (2009). Cleft lip and palate. Lancet 374 (9703), 1773–1785. 10.1016/s0140-6736(09)60695-4 19747722

[B29] Ngan P. Wilmes B. Drescher D. Martin C. Weaver B. Gunel E. (2015). Comparison of two maxillary protraction protocols: tooth-borne versus bone-anchored protraction facemask treatment. Prog. Orthod. 16, 26. 10.1186/s40510-015-0096-7 26303311 PMC4547969

[B30] Nucci L. Costanzo C. Carfora M. d'Apuzzo F. Franchi L. Perillo L. (2021). Dentoskeletal effects of early class III treatment protocol based on timing of intervention in children. Prog. Orthod. 22 (1), 49. 10.1186/s40510-021-00392-2 34935091 PMC8692548

[B31] Park J. H. Bayome M. Zahrowski J. J. Kook Y. A. (2017). Displacement and stress distribution by different bone-borne palatal expanders with facemask: a 3-dimensional finite element analysis. Am. J. Orthod. Dentofac. Orthop. 151 (1), 105–117. 10.1016/j.ajodo.2016.06.026 28024761

[B32] Parveen S. Husain A. Gosla Reddy S. Mascarenhas R. Shenoy S. (2020). Three-dimensional finite element analysis of initial displacement and stress on the craniofacial structures of unilateral cleft lip and palate model during protraction therapy with variable forces and directions. Comput. Methods Biomech. Biomed. Engin 23 (16), 1360–1376. 10.1080/10255842.2020.1803844 32873066

[B33] Santiago P. E. Schuster L. A. Levy-Bercowski D. (2014). Management of the alveolar cleft. Clin. Plast. Surg. 41 (2), 219–232. 10.1016/j.cps.2014.01.001 24607190

[B34] Schmidt H. Alber T. Wehner T. Blakytny R. Wilke H. J. (2009). Discretization error when using finite element models: analysis and evaluation of an underestimated problem. J. Biomech. 42 (12), 1926–1934. 10.1016/j.jbiomech.2009.05.005 19501362

[B35] Shi B. Losee J. E. (2015). The impact of cleft lip and palate repair on maxillofacial growth. Int. J. Oral Sci. 7 (1), 14–17. 10.1038/ijos.2014.59 25394591 PMC4817536

[B36] Tai C. C. Sutherland I. S. McFadden L. (2000). Prospective analysis of secondary alveolar bone grafting using computed tomography. J. Oral Maxillofac. Surg. 58 (11), 1241–1249. 10.1053/joms.2000.16623 11078135

[B37] Tanaka O. M. Saga A. Y. Pithon M. M. Argenta M. A. (2016). Stresses in the midpalatal suture in the maxillary protraction therapy: a 3D finite element analysis. Prog. Orthod. 17, 8. 10.1186/s40510-016-0121-5 26980199 PMC4792831

[B38] Tanne K. Sakuda M. Burstone C. J. (1987). Three-dimensional finite element analysis for stress in the periodontal tissue by orthodontic forces. Am. J. Orthod. Dentofac. Orthop. 92 (6), 499–505. 10.1016/0889-5406(87)90232-0 3479896

[B39] Thresher R. W. Saito G. E. (1973). The stress analysis of human teeth. J. Biomech. 6 (5), 443–449. 10.1016/0021-9290(73)90003-1 4748494

[B40] Tian T. Huang H. Y. Wang W. Shi B. Zheng Q. Li C. H. (2022). Three-dimensional finite element analysis of the effect of alveolar cleft bone graft on the maxillofacial biomechanical stabilities of unilateral complete cleft lip and palate. Biomed. Eng. Online 21 (1), 31. 10.1186/s12938-022-01000-y 35596229 PMC9123812

[B41] Toro-Ibacache V. Cortes Araya J. Diaz Munoz A. Manriquez Soto G. (2014). Morphologic variability of nonsyndromic operated patients affected by cleft lip and palate: a geometric morphometric study. Am. J. Orthod. Dentofac. Orthop. 146 (3), 346–354. 10.1016/j.ajodo.2014.06.002 25172257

[B42] Trojan L. Gonzalez Torres L. A. Las Casas E. B. Melo A. C. Dorneles L. S. (2013). “Strain level at midpalatal suture–Correlation with mechanobiological concepts,” in 22nd International Congress of Mechanical Engineering, Ribeirão Preto, SP, Brazil, November 3-7, 2013.

[B43] Velez-Muriel S. M. Talma E. Romanyk D. L. Las Casas E. B. Guerrero-Vargas J. A. Garzon-Alvarado D. A. (2021). Biomechanical behavior of an alveolar graft under maxillary therapies. Biomech. Model Mechanobiol. 20 (4), 1519–1532. 10.1007/s10237-021-01460-6 33893875

[B44] Wang D. Cheng L. Wang C. Qian Y. Pan X. (2009). Biomechanical analysis of rapid maxillary expansion in the UCLP patient. Med. Eng. Phys. 31 (3), 409–417. 10.1016/j.medengphy.2008.06.011 18760953

[B45] Widmark G. Haraldson T. Kahnberg K. E. (1995). Functional evaluation after TMJ surgery. J. Oral Rehabil. 22 (8), 589–593. 10.1111/j.1365-2842.1995.tb01054.x 7472730

[B46] Yang C. J. Pan X. G. Qian Y. F. Wang G. M. (2012a). Impact of rapid maxillary expansion in unilateral cleft lip and palate patients after secondary alveolar bone grafting: review and case report. Oral Surg. Oral Med. Oral Pathol. Oral Radiol. 114 (1), e25–e30. 10.1016/j.tripleo.2011.08.030 22732853

[B47] Yang I. H. Chang Y. I. Kim T. W. Ahn S. J. Lim W. H. Lee N. K. (2012b). Effects of cleft type, facemask anchorage method, and alveolar bone graft on maxillary protraction: a three-dimensional finite element analysis. Cleft Palate Craniofac J. 49 (2), 221–229. 10.1597/10-265 21740169

[B48] Yepes E. Quintero P. Rueda Z. V. Pedroza A. (2014). Optimal force for maxillary protraction facemask therapy in the early treatment of class III malocclusion. Eur. J. Orthod. 36 (5), 586–594. 10.1093/ejo/cjt091 24351569

[B49] Yoshida N. Koga Y. Peng C. L. Tanaka E. Kobayashi K. (2001). *In vivo* measurement of the elastic modulus of the human periodontal ligament. Med. Eng. Phys. 23 (8), 567–572. 10.1016/s1350-4533(01)00073-x 11719079

[B50] Yu H. S. Baik H. S. Sung S. J. Kim K. D. Cho Y. S. (2007). Three-dimensional finite-element analysis of maxillary protraction with and without rapid palatal expansion. Eur. J. Orthod. 29 (2), 118–125. 10.1093/ejo/cjl057 17218719

[B51] Zhang W. Shen G. Wang X. Yu H. Fan L. (2012). Evaluation of alveolar bone grafting using limited cone beam computed tomography. Oral Surg. Oral Med. Oral Pathol. Oral Radiol. 113 (4), 542–548. 10.1016/j.oooo.2011.10.001 22668433

