# Supplementary material for: Finite element analysis of maxillary orthodontic therapies with variable alveolar bone grafts under occlusal forces in patient with unilateral cleft lip and palate
Source: Front Bioeng Biotechnol. 2024 Nov 5;12:1448286. doi: 10.3389/fbioe.2024.1448286 (PMC11573579; doi:10.3389/fbioe.2024.1448286)
Supplement: Supplementary file 1 [file DataSheet1.PDF]

Supplementary Table 1 Horizontal(x) Displacement Value of the Landmarks the 8 bone graft models under maxillary protraction(mm)

Selected Landmarks		Non-bone graft	Full maxilla cleft	Full alveolar cleft	Lower 2/3	Upper 2/3	Lower 1/3	Middle 1/3	Upper 1/3
non-cleft side	1	-2.20E-02	-8.34E-03	-8.58E-03	-8.37E-03	-7.69E-03	-7.42E-03	-7.35E-03	-7.74E-03
	2	-2.38E-02	-8.40E-03	-8.76E-03	-8.61E-03	-8.10E-03	-7.98E-03	-7.92E-03	-8.28E-03
	3	-2.92E-02	-8.72E-03	-9.09E-03	-9.07E-03	-8.95E-03	-9.47E-03	-9.16E-03	-9.66E-03
	4	-4.33E-02	-4.01E-02	-4.07E-02	-4.09E-02	-4.08E-02	-4.11E-02	-4.12E-02	-4.12E-02
	5	-9.20E-03	-7.20E-03	-7.78E-03	-7.18E-03	-6.10E-03	-4.15E-03	-4.81E-03	-5.49E-03
	6	-1.05E-02	-7.19E-03	-8.22E-03	-7.69E-03	-6.75E-03	-5.02E-03	-5.61E-03	-6.29E-03
	7	-1.54E-02	-6.67E-03	-8.09E-03	-7.77E-03	-7.17E-03	-6.21E-03	-6.50E-03	-7.07E-03
	8	-2.06E-02	-5.48E-03	-8.64E-03	-8.65E-03	-8.67E-03	-8.75E-03	-8.68E-03	-9.23E-03
cleft side	4	2.76E-02	2.77E-02	2.72E-02	2.72E-02	2.75E-02	2.79E-02	2.76E-02	2.73E-02
	5	-5.94E-03	-5.98E-03	-1.16E-02	-1.23E-02	-1.60E-02	-1.44E-02	-1.66E-02	-1.98E-02
	6	-3.71E-03	-4.44E-03	-9.11E-03	-9.78E-03	-1.31E-02	-1.17E-02	-1.37E-02	-1.66E-02
	7	-2.41E-03	-7.04E-03	-1.11E-02	-1.15E-02	-1.43E-02	-1.21E-02	-1.45E-02	-1.72E-02
	8	3.94E-03	-7.35E-03	-7.44E-03	-7.29E-03	-8.49E-03	-6.13E-03	8.19E-03	-9.45E-03

Supplementary Table 2 Sagittal(y) Displacement Value of the Landmarks the 8 bone graft models under maxillary protraction(mm)

Selected Landmarks		Non-bone graft	Full maxilla cleft	Full alveolar cleft	Lower 2/3	Upper 2/3	Lower 1/3	Middle 1/3	Upper 1/3
non-cleft side	1	-2.68E-02	-2.38E-02	-2.65E-02	-2.68E-02	-2.71E-02	-2.80E-02	-2.78E-02	-2.76E-02
	2	-2.57E-02	-2.22E-02	-2.53E-02	-2.56E-02	-2.61E-02	-2.71E-02	-2.68E-02	-2.67E-02
	3	-2.19E-02	-1.81E-02	-2.17E-02	-2.22E-02	-2.27E-02	-2.38E-02	-2.36E-02	-2.33E-02
	4	-1.13E-02	-1.53E-02	-1.46E-02	-1.43E-02	-1.44E-02	-1.35E-02	-1.40E-02	-1.40E-02
	5	-3.56E-02	-3.74E-02	-3.54E-02	-3.51E-02	-3.47E-02	-3.44E-02	-3.43E-02	-3.40E-02
	6	-3.25E-02	-3.65E-02	-3.32E-02	-3.27E-02	-3.19E-02	-3.11E-02	-3.12E-02	-3.08E-02
	7	-2.73E-02	-3.12E-02	-2.80E-02	-2.76E-02	-2.68E-02	-2.61E-02	-2.62E-02	-2.58E-02
	8	-2.60E-02	-2.97E-02	-2.65E-02	-2.62E-02	-2.53E-02	-2.47E-02	-2.48E-02	-2.45E-02
cleft side	4	-2.60E-03	-1.56E-03	-2.80E-03	-2.85E-03	-2.83E-03	-2.20E-02	-2.85E-03	-3.20E-03
	5	-2.66E-02	-2.78E-02	-2.75E-02	-2.74E-02	-2.69E-02	-2.69E-02	-2.68E-02	-2.65E-02
	6	-2.16E-02	-2.52E-02	-2.26E-02	-2.23E-02	-2.09E-02	-2.11E-02	-2.06E-02	-1.95E-02
	7	-1.40E-02	-1.89E-02	-1.55E-02	-1.51E-02	-1.37E-02	-1.39E-02	-1.33E-02	-1.22E-02
	8	-1.34E-02	-1.87E-02	-1.41E-02	-1.37E-02	-1.23E-02	-1.27E-02	-1.19E-02	-1.06E-02

Supplementary Table 3 The number of elements and nodes obtained by tetrahedral meshing of 8 bone graft models in ANSYS Workbench

model		elements	nodes
**protraction without expansion**	**Non-bone graft**	412918	801620
	**Full maxilla cleft**	417512	810368
	**Full alveolar cleft**	414564	804679
	**Lower 2/3**	414436	804488
	**Upper 2/3**	414261	804159
	**Lower 1/3**	413750	803262
	**Middle 1/3**	413634	803068
	**Upper 1/3**	413552	802902
**expansion only**	**Non-bone graft**	405515	795106
	**Full maxilla cleft**	410688	805270
	**Full alveolar cleft**	407731	799572
	**Lower 2/3**	407078	798250
	**Upper 2/3**	406923	797953
	**Lower 1/3**	406323	796725
	**Middle 1/3**	406270	796631
	**Upper 1/3**	406168	796428
**protraction with expansion**	**Non-bone graft**	492853	939980
	**Full maxilla cleft**	497447	948728
	**Full alveolar cleft**	494499	943039
	**Lower 2/3**	494371	942848
	**Upper 2/3**	494196	942519
	**Lower 1/3**	493685	941622
	**Middle 1/3**	493569	941428
	**Upper 1/3**	493487	941262

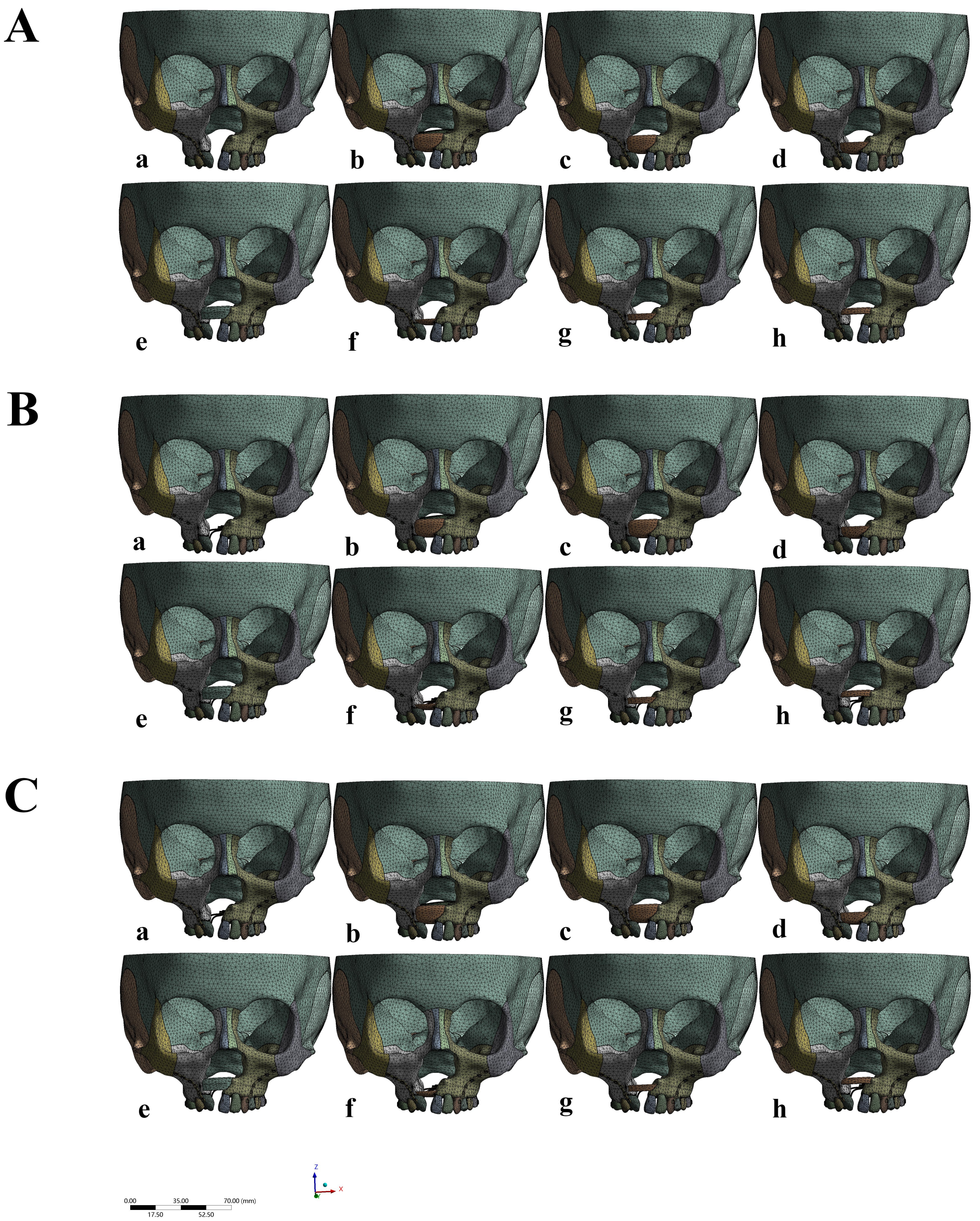

Supplementary Figure 1 Tetrahedral meshing results of models. **A** Working conditions of protraction without expansion; **B** Working conditions of expansion only; **C** Working conditions of protraction with expansion. Tetrahedral meshing results of non-bone graft model (a) and full maxilla cleft (b), full alveolar cleft (c), lower 2/3 (d), upper 2/3 (e), lower 1/3 (f), middle 1/3 (g), upper 1/3 (h) bone graft model.
